# Effects of Suprasegmental Phonological Alternations on Early Word Recognition: Evidence from Tone Sandhi

**DOI:** 10.3389/fpsyg.2016.00627

**Published:** 2016-05-03

**Authors:** Thilanga D. Wewalaarachchi, Leher Singh

**Affiliations:** Department of Psychology, National University of Singapore, SingaporeSingapore

**Keywords:** phonological alternation, Sandhi, lexical representation, lexical tone, language acquisition

## Abstract

Early language acquisition is potentially complicated by the presence of many sources of variability in the speech signal. A frequent example of variability is phonological alternations, which can lead to context-driven changes in the realization of a word. The aim of the current study was to investigate effects of a highly frequent yet scarcely researched type of suprasegmental phonological alternation – tone Sandhi – on early spoken word recognition. The tone Sandhi rule investigated herein involves a tone change of the first syllable in a disyllabic unit. In accordance with third tone Sandhi, when two dipping tone syllables are juxtaposed in connected speech, the first syllable is dissimilated to a high rising tone. For example, ‘flour mill’ (unaltered pre-Sandhi form [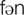
(214) 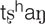
(214)]) undergoes tonal alternation resulting in the altered post-Sandhi form [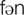
(35) 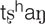
(214)]. In the current study, preschoolers’ sensitivity to the effects of tone Sandhi when processing familiar words was investigated via a preferential looking paradigm. Words varied in their phonological form: one set of words was labeled with a phonological alternation due to Sandhi (Post Sandhi), one set of words was labeled with an unaltered Sandhi form (Pre Sandhi), one set consisted of non Sandhi words (Correct Pronunciation, and one set were labeled with a tonal alternation not associated with Sandhi rules (Mispronunciation). Post-Sandhi forms and correct pronunciations were associated with visual referents with comparable strength, with only a subtle processing cost observed for post-Sandhi forms in the time course of lexical selection. Likewise, pre-Sandhi forms and true mispronunciations were rejected as labels for visual references with comparable strength, with only subtle differences observed in the time course of lexical selection. Findings are discussed in terms of their impact on prevailing theories of lexical representation.

## Introduction

In order to learn the meanings of words, children must possess a clear understanding of how words are phonologically defined in their native language. However, there is a wide range of natural phonetic variation contained within the speech signal. This variation can complicate the pathway to accurate phonological definitions. Significant variation derives from constraints on how phonemes are realized when placed in sequence. For example, when producing the word *‘handbag’* [hændbæg] in natural discourse, a probable acoustic realization is the alternating form *‘hambag’* [hæmbæg]. As a result of the phonological processes of assimilation and elision, the phoneme [d] is omitted and the phoneme [n] is assimilated to the phoneme [m], creating a phonological alternation. A young child must appreciate that the resulting form [hæmbæg] is lexically equivalent to its unaltered form [hændbæg], thereby mitigating the effects of the alternation. Phonological alternations are a universal feature of human languages and must be successfully negotiated by young language learners in their journey toward native language proficiency.

While there have been several investigations of how young children contend with phonological alternations (e.g., Skoruppa et al., 2013a,b), these investigations have focused exclusively on consonantal changes belonging to consonant-vowel phonological systems such as English and French. However, the majority of the world’s language learners master a native language with three sources of phonemic variation: consonants, vowels and lexical tone (Yip, 2002). Tone languages also contain phonological alternations. Most notably, they incorporate a particular form of phonological alternation that results in a whole-tone substitution in particular contexts (Chao, 1951). This type of phonological alternation, termed tone Sandhi, is a highly frequent source of lexical ambiguity in human languages (Chen, 2000), yet there have been no empirical studies on children’s ability to contend with variability introduced by Sandhi phenomena in language comprehension. The purpose of the current study was to investigate children’s ability to negotiate tone Sandhi in a crucial component of language development: spoken word recognition.

The most widely studied tone Sandhi phenomena^1^ in Mandarin Chinese is the third tone Sandhi rule (Chen, 2000). Mandarin Chinese has four lexical tones: high level tone (55), high rising tone (35), dipping tone (214), and falling tone (51). Mandarin tones are standardly defined by the Chao numeral system used hereafter. This system marks each tone-bearing syllable according to its relative onset/offset pitch (and medial pitch for the bidirectional dipping tone). According to this system, a value of 5 represents the highest lexical pitch and 1 represents the lowest lexical pitch (Chao, 1951). In accordance with the third tone Sandhi rule, when two syllables each produced in dipping tone occur next to each other in connected speech, the first syllable undergoes tonal alternation and changes to high rising tone. This is an example of a tonal dimissilation process, where a given tone is made less similar to the tone adjacent to it (Lin, 2007). Third tone Sandhi is construed as phonological neutralization in that a dipping tone syllable that has undergone tonal alternation is non-contrastive with an unaltered high rising tone syllable. In other words, native speakers cannot perceptually differentiate an unaltered high rising tone from an alternating dipping tone that has undergone this particular tonal alternation (Peng, 2000). It has been proposed that tone Sandhi eases tone perception in view of the fact that the tonal transition between high rising(35)/dipping(214) syllables is easier to detect than the tone transition between dipping(214)/dipping(214) syllables (Lin, 2007). However, this process presents a potentially serious challenge for learners: ignoring the tone alternation can lead to a phonologically illegal interpretation, yet encoding the alternating form – without compensating for the effects of the Sandhi rule – can lead to a lexical ambiguity. For example, Sandhi rules dictate that the phrase/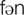
(214) 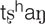
(214)/ (flour mill; 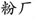) is obligatorily modified such the first syllable is alternated to [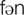
(35) 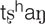
(214)], whilst preserving the original meaning of the word. However, given that the /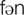
(35) 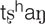
(214)/ means ‘graveyard’ (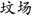), this tonal alternation creates a potential lexical ambiguity (Chen, 2000). Listeners have to therefore recognize that tone substitutions due to Sandhi (or post-Sandhi forms) preserve the same meaning as pre-Sandhi forms, while recognizing that the latter is a phonologically illegal tone sequence.

While there has been no experimental research on children’s abilities to process post-Sandhi forms in language comprehension, researchers have documented children’s application of third tone Sandhi rules in elicited speech production (e.g., Chen et al., 2010; Wang, 2011). These studies have revealed that children between 3 and 5 years of age demonstrate a sustained period of uncertainty when applying third tone Sandhi rules in production (Chen et al., 2010; Wang, 2011). Over this period, children face difficulties applying third tone Sandhi rules to words in production tasks (Wang, 2011). In prior research, 6 years of age serves as a ‘tipping point’ after which children’s knowledge of tone Sandhi solidifies and Sandhi rules are reliably applied in production (Wang, 2011). On account of this developmental timeline, the current study investigates whether the comprehension of Sandhi forms between the ages of 3 and 5 years is equally fragile or whether in the domain of comprehension, Sandhi forms appear representationally intact.

Psycholinguistic theories offer competing proposals with regards to how Sandhi can be accounted for in language processing, varying notably in terms of scope and specificity of information stored in tonal representations (e.g., Lahiri and Marslen-Wilson, 1992; Ohala and Ohala, 1995; Gaskell and Marslen-Wilson, 1996). There are three primary accounts of how Sandhi forms are represented in memory: the surface view, the canonical view, and the underspecification view. The surface view proposes that words are stored in their encountered (or surface) form (the post-Sandhi form) and that listeners apply a set of learned rules to recode alternating forms into underlying forms (Ohala and Ohala, 1995; Gaskell and Marslen-Wilson, 1996). Therefore, the surface account would predict that the word ‘flour mill’ [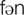
(35) 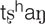
(214)] is represented as post-Sandhi form /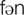
(35) 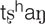
(214)/ and not as its underlying pre-Sandhi form /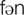
(214) 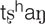
(214)/. According to the surface account, although words are stored as encountered, an additional mechanism then mediates between speech input and underlying forms. An additional mechanism is required because alternations can result in activation of two lexical candidates. This ambiguity is then resolved via post-lexical processes, such as context-driven inferences that are then applied to phonological output (e.g., Gaskell and Marslen-Wilson, 1996). The second view – the canonical view – is that words are stored in their citation or canonical form (pre-Sandhi form) even though they are encountered as post-Sandhi forms. This view posits a mechanism whereby post-Sandhi forms are re-written regressively after the onset of the second syllable to match the underlying stored representation (Zhou and Marslen-Wilson, 1997). This mechanism is activated in Sandhi contexts, where the syllable directly adjacent to a high rising tone is a dipping tone (Zhou and Marslen-Wilson, 1997). Therefore, the canonical account would predict that ‘flour mill’ [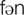
(35) 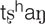
(214)] is ultimately represented in its pre-Sandhi form /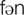
(214) 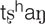
(214)] / and that it is re-written after the onset of the second syllable to match the stored representation, the pre-Sandhi form. A third possibility derives from theories of phonological underspecification (e.g., Lahiri and Marslen-Wilson, 1991). According to these theories, words are defined in the lexicon by phonological features. Values are linked to these features (e.g., presence of voicing). Feature values vary in the extent to which they are specified. Phonological features that remain consistent across contexts are fully specified. By contrast, feature values that change albeit predictably, as in the case of phonological alternations, are underspecified and are weakly represented in the lexicon. Therefore, the underspecification account would predict that ‘flour mill’ [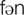
(35) 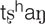
(214)] is represented as [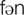
(under-specified for tone) 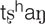
(214)] and that both [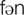
(35)] and [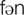
(214)] are initially activated upon hearing this word. As such, in the case of an alternation leading to lexical ambiguity (e.g., /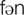
(214) 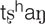
(214)/ and /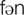
(35) 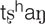
(214)/, both candidates (i.e., [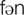
(35)] and [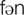
(214)]) are activated. As with surface theories, post-lexical processes (e.g., contextual cues, semantic plausibility, and statistical inferences) then allow for the deactivation and eventual rejection of the incorrect candidate.

Theories that presume storage of surface forms or canonical forms versus those that rely on underspecification of phonetic feature values notably differ in the level of abstraction they impute to lexical representations: the former theories (surface view and canonical view) presume that words are stored at the level of exemplars and exemplars are then matched with input, whereas the latter theory (underspecification) presumes more abstract representations that flexibly represent particular phonetic details based on how robustly they are contrasted within a language. All of these theories, however, derive principally from evidence drawn from consonantal alternations and also from adult language processing. Although the current study does not explicitly adjudicate between these theories, understanding the development of children’s ability to contend with alternations, particularly suprasegmental alternations, could enrich the evidence basis on which prevailing theories are founded. Tone Sandhi is a highly frequent phonological process in human languages (see Gandour, 1978), yet Sandhi processing remains largely unaccounted for in extant theories of lexical representation (but see Zhou and Marslen-Wilson, 1997).

The goal of the current study was to launch an experimental investigation into young children’s abilities to process tone Sandhi forms in spoken word recognition. Preschool children were presented with familiar words that assumed four possible forms: words that were labeled with a phonological alternation due to Sandhi (Post-Sandhi); words that were labeled with an unaltered form when tone Sandhi was licensed (Pre-Sandhi); non-Sandhi words correctly produced (Correct Pronunciation); and words labeled with a phonological alternation of tone not associated with Sandhi rules (Mispronunciation). Children’s capacity to recognize familiar words was evaluated under each of these conditions.

Prevailing psycholinguistic theories offer different predictions about how Sandhi forms would be processed relative to correct pronunciations and mispronunciations, which can be examined in the current study. These accounts make predictions that can be examined in two ways in the current study where familiar word recognition is investigated. First, responses to Sandhi forms can be examined by tracking aggregate responses (i.e., does the child map a pre- or post-Sandhi form onto a visual target or not?). These responses are usually determined by the likelihood of children preferentially fixating a visual target over a distractor object upon hearing a verbal label. Second, an additional measure derives from the time course of fixation patterns. In other words, how quickly does a participant shift his/her gaze to a visual target upon hearing an auditory label? For this reason, the current study also examines time-dependent measures of activation upon hearing auditory labels. Predictions invited by each model for the current study are described in succession and summarized in **Table 1**. First, the surface view predicts greater efficiency in processing post-Sandhi forms relative to pre-Sandhi forms. In a preferential looking paradigm, this would be hypothesized to lead to rapid rejection of pre-Sandhi forms as labels for visual targets and rapid acceptance of post-Sandhi forms as acceptable labels for visual targets. According to the surface view, the time course of processing post-Sandhi forms would mirror that of correct pronunciations, with both types of words associated with equivalent visual orientation toward the target object. Additionally, the surface view predicts that the time course of processing pre-Sandhi forms would be similar to that of processing mispronunciations, both resulting in an absence of visual preference for the target or distractor object. In contrast, the canonical view predicts late recognition of post-Sandhi forms following the onset of the second syllable relative to correct pronunciations. Unlike post-Sandhi forms, correct pronunciations do not require regressive re-writing to match the stored representation. Although not explicitly stated, an additional tacit assumption of the canonical view is early recognition of pre-Sandhi forms, which correspond to the stored representation for Sandhi words. Therefore, in line with the canonical view, the time course of processing pre-Sandhi forms would mirror that of correct pronunciations, with both pronunciation types associated with a preference for the target object. The canonical view – which is better informed by time course analyses – would predict a late-emergent target preference in response to post-Sandhi forms. This view predicts that the time course of processing post-sandhi and mispronunciations would initially result in similar fixation patterns (during the first syllable), but that fixation patterns to post-Sandhi forms would diverge from mispronunciations after the onset of the second syllable. Lastly, the underspecification view predicts comparable efficiency in processing both post-Sandhi and pre-Sandhi forms. According to the underspecification view, pre-Sandhi, post-Sandhi and correct pronunciations would exhibit similar fixation patterns, with the time course of processing all three pronunciation types indicating incremental shifts toward the target object. This view would predict convergent responses to pre- and post-Sandhi forms early in the processing window followed by preferential fixation to the target late in the processing window as Sandhi forms are putatively resolved via post-lexical processes.

**Table 1:** Summary of predicted findings for surface, canonical and underspecified models.

Account	Stored representations	Predictions
Surface	Post-Sandhi	Target preference in response to post-Sandhi forms No target preference in response to pre-Sandhi forms Time course of processing post-Sandhi forms mirrors correct pronunciations: incremental shifts to target Time course of processing pre-Sandhi forms mirrors mispronunciations: sustained distractor preference
Canonical	Pre-Sandhi	Late target preference in response to post-Sandhi based on re-writing to pre-Sandhi form after onset of second syllable. Early target preference in response to pre-Sandhi form. Time course of processing pre-Sandhi forms mirrors correct pronunciations: incremental shifts to target Time course of processing post-Sandhi forms initially mirrors mispronunciations but shows late divergence, increasing shifts toward the target after onset of second syllable
Underspecified	Abstract	Target preference in response to post-Sandhi form Target preference in response to pre-Sandhi form Time course of processing pre-Sandhi and post-Sandhi forms mirrors correct pronunciations: incremental shifts to target

## Materials and Methods

### Participants

Forty-nine native speakers of Mandarin were sampled between 3 to 5 years old (range: 36–60 months; mean: 47 months). Three additional children were tested but data were excluded due to inattention and failure to provide complete data. None of the participants had any known developmental delays or disabilities.

### Stimuli

#### Auditory Stimuli

Twelve concrete, imageable, disyllabic nouns that were judged to be familiar to children aged 3–5 were chosen as test stimuli (see **Table 2** for a list of target words). The number of items was necessarily limited by the availability of Sandhi forms with which children were likely to be familiar. In order to confirm that these words were indeed understood by the children sampled, a receptive vocabulary test was conducted after the study was completed. Familiar objects were paired with each other and participants were asked to point to the target. Each test item began with the question, “
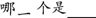
” ([na(214) i(55) 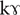
(51) 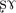
(51)]; translates to: which [one] is the ___?). Incorrect and null responses were taken to mean that the child did not know the meaning of the word tested, and were excluded from subsequent analysis for individual participants. Participants scored an average of 99.3% on a test of their knowledge of the meanings of Correct Pronunciation words (*SD* = 0.34) and 99.5% on post-Sandhi forms (*SD* = 0.24).

**Table 2:** Target words and referents (in parentheses).

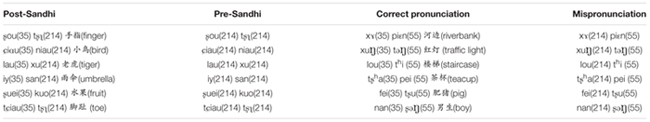

During the experiment, there were four types of stimuli used. The first type comprised words where Sandhi was licensed and applied correctly (Post-Sandhi). Within the first type, two dipping tone syllables were juxtaposed, but the first syllable was appropriately alternated with a high rising form. The second type comprised words where Sandhi was licensed but not applied (Pre-Sandhi). Within the second type, two dipping tone syllables occurred in succession. This is an omission of Sandhi rules, as the first syllable would typically be alternated with a high rising form. The third type comprised words that were not subject to Sandhi rules and were correctly pronounced (Correct Pronunciation). Within the third type, the correct tone assignment was always a high rising tone (first syllable) followed by a high level tone (second syllable). In the final trial type, words that underwent phonological alternations that were not linked to Sandhi (Mispronunciation) were included. Within the final type, the first syllable was substituted with a dipping tone (i.e., originally high rising tone), while the second syllable remained unchanged (i.e., high level tone). All target words were embedded in final position of the carrier phrase, “
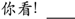
” ([ni(214) k^h^an(51)]; translates to: Look! ___).

A female undergraduate student who was a native speaker of Mandarin recorded the speech stimuli for the study in a soundproof recording booth. Stimuli were produced in infant-directed register. Since the words in the pre-Sandhi condition were illegal in Mandarin and therefore potentially unnatural to produce, a list of disyllabic words with a homophonic first syllable that corresponded to the first syllable of the words in the pre-Sandhi condition was recorded instead. The first syllable of this list was spliced and digitally inserted to replace the first syllable in the post-Sandhi condition to create stimuli for the pre-Sandhi condition.

#### Stimulus Validation

**FIGURE 1:**
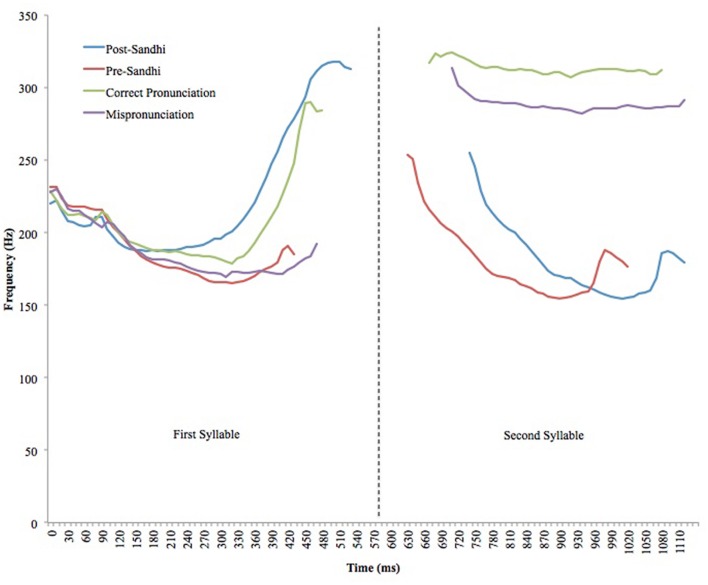
**Sample pitch contours of post-Sandhi, pre-Sandhi, correct, and mispronounced forms**.

Acoustic analyses were conducted to ensure that the stimuli conveyed the desired tone. The average values for mean pitch, minimum pitch, maximum pitch; onset and duration was calculated for each syllable in all four conditions (Post-Sandhi, Pre-Sandhi, Correct Pronunciation, Mispronunciation) (see **Table 3** for results of acoustic analysis)^2^. Mean, minimum and maximum pitch values for the high level, high rising and dipping tones were then calculated to ensure that the syllables were tonally contrastive as intended. Dipping tone syllables had the lowest mean minimum pitch (187 Hz) and high rising syllables had the highest mean maximum pitch (311 Hz). This follows the expected pitch contour pattern for Mandarin tones (see **Figure 1** for sample pitch contours of all tones within the stimulus set).

**Table 3:** Acoustic analyses for target words.

Condition	Tone	Mean pitch (Hz)	Minimum pitch (Hz)	Maximum pitch (Hz)	Onset (Hz)	Duration (ms)
Post-Sandhi	First syllable	2	240	195	349	229	624
	Second syllable	3	190	105	274	269	564
Pre-Sandhi	First syllable	3	184	163	232	230	573
	Second syllable	3	183	103	264	261	564
Correct pronunciation	First syllable	2	206	179	273	218	577
	Second syllable	1	290	280	309	300	536
Mispronunciation	First syllable	3	183	168	227	624	219
	Second syllable	1	259	259	301	292	615

In order to confirm that tone identity was clearly conveyed to the native ear, ten adult native speakers of Mandarin (mean age = 22.60 years, *SD* = 0.97) were presented with the first syllables from each condition (Post-Sandhi, Pre-Sandhi, Correct Pronunciation and Mispronunciation) and asked to categorize the tones as high level, high rising, dipping, falling or unsure. The raters were able to identify all tones at a mean accuracy of 94%, suggesting that adult speakers were able to make accurate judgments between high rising and dipping tones.

### Procedure

**FIGURE 2:**
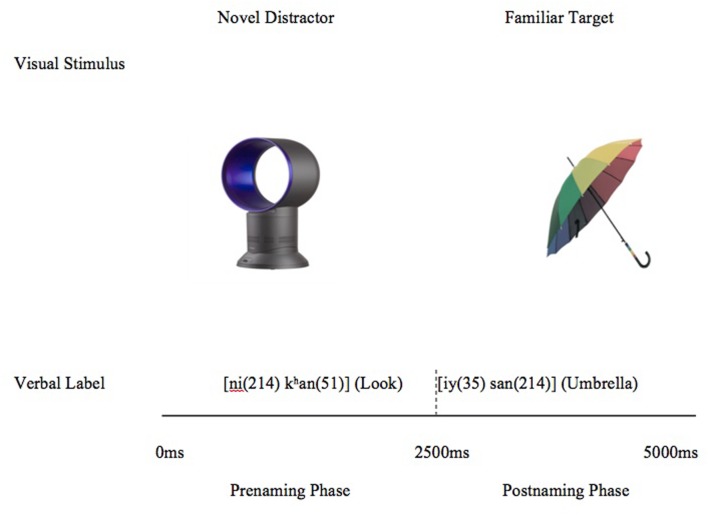
**Sequence of events in each experimental trial**.

The Preferential Looking Paradigm was employed to assess spoken word recognition as in prior investigations (e.g., Bailey and Plunkett, 2002; Ballem and Plunkett, 2005; Mani and Plunkett, 2010). All children were tested in a quiet room with a caregiver present. Children sat approximately 30 cm from a Macintosh computer, which was placed at eye-level. Auditory stimuli were transmitted via external speakers at a conversational level of approximately 70 dB. The experiment began with three training trials where two familiar objects were presented side by side accompanied by auditory stimuli. This served to familiarize the child with the experimental paradigm. Immediately following the training trials, participants viewed 24 test trials in a randomized order. The trials had two phases – the pre-naming phase and the post-naming phase, each lasting 2500 ms. Trials began with 1000 ms of silence followed by the disyllabic directive,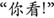 (English translation: Look!). The duration of the directive 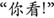 was 900 ms. Following this, there was 600 ms of silence, followed by the onset of the target word (exactly 2500 ms after trial onset). This marked the start of the post-naming phase. Visual stimuli comprised of photographs of target words and unfamiliar objects that served as distractor foils (see **Figure 2** for a sample trial). Unfamiliar objects were chosen in the present study as the presence of unfamiliar object provides a potential alternative referent for a mispronounced form (see Singh et al., 2015, for a comparison of naming effects for familiar and unfamiliar distractors). Unfamiliar distractors were also chosen on account of the fact that familiar distractors may lead to a scenario where word recognition is constrained by similarity of the target label to the auditory stimulus and/or by the dissimilarity of the distractor label to the auditory stimulus. These routes to lexical selection are hard to differentiate (see White and Morgan, 2008 for a discussion of these issues) and as such, unfamiliar distractors were determined to be preferable for our design.

The distractors were semantically and phonologically unrelated to the targets. The visual display stayed on the screen for the entire duration of 5000 ms. The sequence of trial types was randomized and side of presentation of target object was counterbalanced. Target words lasted an average of 1068 ms (range 850–1188 ms). Target fixations to the visual display were coded oﬄine using SuperCoder (Hollich, 2005) for the entire duration of the trial. Coders determined for each trial, frame-by-frame (at a frame rate of 30 frames per second), whether children were fixating on the target or distractor. An independent coder coded 25% of the participant videos and inter-rater reliability was.96. As with prior studies investigating children’s responses to mispronunciations, eye-gaze data was used to evaluate the accuracy and efficiency of spoken word recognition.

Dependent measures consisted of naming effects and time course analyses. Naming effects were computed by subtracting fixation times during the pre-naming phase from fixation times during the post-naming phase. A significant elevation in fixation between pre- and post-naming typically provides evidence that children have mapped an auditory label onto the visual target. A significant decrease in fixation between pre- and post-naming typically indicates that participants have preferentially fixated upon the distractor object, possibly associating the verbal label with the distractor object, forming a novel mapping. No significant change across pre- and post-naming phases suggests ambiguity in how verbal labels correspond to visual targets (Schafer and Plunkett, 1998; Meints et al., 1999; Swingley and Aslin, 2000; Bailey and Plunkett, 2002).

In addition to naming effects, the time course of spoken word recognition was analyzed in order to evaluate the efficiency with which word selection occurred upon hearing a verbal label. Time course analyses go beyond aggregate fixation to the target, but rather, chart frame-by-frame the pattern of fixation obtained for each trial type after the presentation of a verbal label. This type of analysis has the potential to reveal more nuanced differences in fixation patterns for different trial types that may be obscured by aggregate measures (see Fernald et al., 2001). This study was carried out in accordance with the recommendations of the Institutional Review Board, National University of Singapore. The protocol was approved by the Institutional Review Board, National University of Singapore.

## Results

An initial set of analyses focused on naming effects for which the dependent measure was the difference in proportion total looking to target (PTL) between the pre-naming and the post-naming phase. PTL is conventionally defined as T/(T + D); where T is the proportion of total looking time to the target, and T + D is the total looking time to both the target and distracter combined. Naming effects were calculated for the four stimuli types: Post-Sandhi, Pre-Sandhi, Correct Pronunciation and Mispronunciation. Individual trials were excluded from analysis if the child did not fixate to both the target and distracter during the pre-naming phase, if the child had zero attention to the screen during the post-naming phase or if the child did not know the meaning of the word at post-experiment vocabulary test. This resulted in a total of 16% of test trials being excluded.

**FIGURE 3:**
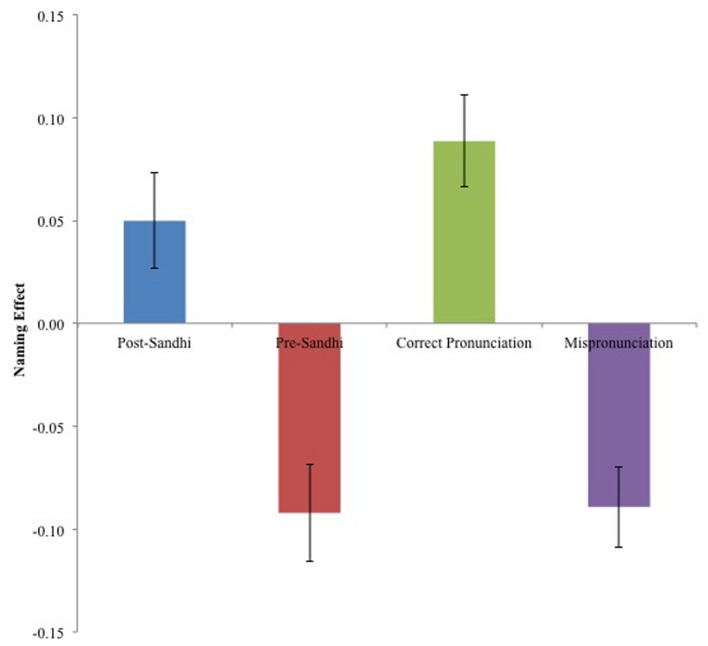
**Naming effects of post-Sandhi, pre-Sandhi, correct, and mispronounced forms (Error bars reflect SEM)**.

Naming effects for each trial type are displayed in **Figure 3**. A 4 × 1 (Trial Type) repeated-measures ANOVA was conducted with naming effects as the dependent measure. Trial type comprised of four levels: post-Sandhi trials, pre-Sandhi trials, Correct Pronunciation trials and Mispronunciation trials. There was a significant main effect of trial type, *F*(1,144) = 16.49, *p* < 0.0001 ($\eta_{p}^{2}$ = 0.26), revealing that naming effects differed by trial type. In order to ascertain the conditions under which participants mapped auditory labels to visual targets, planned comparisons were then conducted to determine whether naming effects departed significantly from zero for each trial type (e.g., Ballem and Plunkett, 2005; Mani and Plunkett, 2007; Mani et al., 2008). Recall that a significant elevation from the pre-naming to post-naming phase (i.e., positive naming effect), indicates that participants have mapped the verbal label onto the referent; a significant decrease indicates a mapping to the distractor (i.e., negative naming effect); and no change (i.e., no naming effect), indicates that neither target nor distractor were associated with the verbal label. A series of one-sample *t*-tests were computed comparing naming effects to zero for each trial type. These comparisons revealed that participants preferentially fixated the target when they heard correct pronunciations and when they heard post-Sandhi forms [*t*(48) = 3.94, *p* < 0.0001, Cohen’s *d* = 0.62, and *t*(48) = 2.2, *p* = 0.03, Cohen’s *d* = 1.14 respectively]. When participants were presented with pre-Sandhi forms and with mispronunciations, however, they demonstrated a significant distractor preference [*t*(48) = -3.89, *p* < 0.0001, Cohen’s *d* = -1.12], and [*t*(48) = -4.45 *p* < 0.0001, Cohen’s *d* = -1.31 respectively]. This suggests that post-Sandhi forms and correct pronunciations were mapped onto visual targets. Both pre-Sandhi forms and true mispronunciations on the other hand, were rejected as possible labels for the target and instead, were mapped onto the distractor. In order to determine whether the magnitude of naming effects statistically differed between post Sandhi words and correct pronunciations, *post hoc* comparisons were computed between post-Sandhi trials and correct pronunciation trials as well as between pre-Sandhi trials and mispronunciation trials. Pairwise comparisons (with Bonferroni corrections) revealed that naming effects obtained did not significantly differ between post-Sandhi trials and correct pronunciation trials or between pre-Sandhi trials and mispronunciations. This suggests that children accepted post-Sandhi forms and correct pronunciations as target referents in equal measure and rejected pre-Sandhi forms and mispronunciations as target referents in equal measure.

A second set of analyses then focused on the time course of spoken word recognition. Unlike analyses of naming effects, which provide insight into children’s preference of one object over the other as a referent, time course analyses illuminate the process by which words are recognized as speech unfolds (Fernald et al., 2008). Naming effects analyses allow inferences to be made about children’s acceptance or rejection of labels as appropriate names for visual targets, while time course analyses afford a more fine-grained view into the constraints on spoken word recognition (Swingley, 2011). The results of time course analyses may qualify conclusions about children’s readiness to accept or reject a label. The use of time course analyses in conjunction with naming effects analyses therefore allows for a more nuanced understanding of some of the constraints on word recognition. In particular, time course analyses have the potential to guide conclusions about whether differences in children’s target and distractor preference emerge early during the processing window, late in the processing window, or even retroactively (e.g., Swingley and Aslin, 2000). This is particularly relevant to the current investigation given that theoretical models of tone Sandhi – in particular, the canonical and underspecification accounts – make different predictions that can be examined by analyzing time course of activation for pre- and post-Sandhi forms.

**FIGURE 4:**
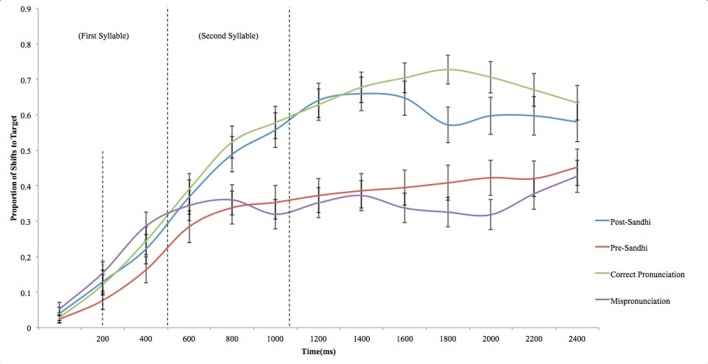
**Proportion of shifts to the target on distractor-first trials (Error bars reflect SEM)**. Dashed lines indicate offset of time allowance provided to launch an eye-movement, average offset of first syllable and average offset of second syllable respectively.

The dependent measure for time-course analysis is typically the proportion of trials for which shifts occur from distractor to target image (Fernald et al., 2001). Only trials on which participants fixated upon the distractor when the verbal label was produced were included in this analysis (see **Figure 4**). **Figure 4** depicts changes in fixation to the target in response to the verbal label. Theoretical accounts of phonological alternation make different predictions with regards to the time course of word recognition of Sandhi forms.

To statistically examine the time course of word recognition, time course data was submitted to repeated-measures ANOVA. Time course data were first aggregated within 200 ms time epochs (e.g., Creel et al., 2006; Louwerse and Bangerter, 2010) after the first 200 ms, resulting in a total of 11 epochs. Customarily, a 200 ms allowance is provided after the onset of the target word to allow for the time required to launch an eye movement to the target (Dahan and Tanenhaus, 2005). A repeated-measures ANOVA with trial type as within-subjects factor and epoch as between-subjects factor was then conducted with proportion of shifts to target as dependent measures. Trial type comprised four levels: post-Sandhi trials, pre-Sandhi trials, Correct Pronunciation trials and Mispronunciation trials. There was a significant main effect of trial type, *F*(3,165) = 3398.82, *p* < 0.0001 ($\eta_{p}^{2}$ = 0.98), revealing that time course of word recognition statistically differed depending on trial type. In addition, there was a significant main effect of epoch, *F*(10,55) = 221.44, *p* < 0.0001 ($\eta_{p}^{2}$ = 1), revealing that time course of word recognition statistically differed depending on epoch. Lastly, there was a significant interaction effect of trial type × epoch, *F*(30,165) = 116.70, *p* < 0.0001 ($\eta_{p}^{2}$ = 0.95), revealing that the differential time course patterns observed for trial types varied systematically depending on epoch. *Post hoc* comparisons were then conducted to statistically examine these effects. For each epoch, *post hoc* comparisons were computed between post-Sandhi trials and pre-Sandhi trials, post-Sandhi trials and correct pronunciation trials as well as between pre-Sandhi trials and mispronunciation trials. Pairwise comparisons revealed a greater proportion of shifts to the target in post-Sandhi trials as compared to the pre-Sandhi trials for all 11 epochs (200–400; 400–600; 600–800; 800–1000; 1000–1200; 1200–1400; 1400–1600; 1600–1800; 1800–2000; 2000–2200; 2200–2400) (*p* < 0.0001) (all contrasts remained significant following Bonferroni correction). This is aligned with results from naming effects, attesting to rejection of the target in response to pre-Sandhi forms and acceptance of the target in response to post-Sandhi forms. However, these findings add to findings gleaned from naming effects by demonstrating that acceptance and rejection of post- and pre-Sandhi forms respectively occur early and endure throughout the processing window.

Further comparisons focused on comparison on shifts to targets for the two types of correct pronunciations (post Sandhi vs. correct pronunciations) and for the two types of mispronunciations (pre Sandhi vs. mispronunciations). The purpose of this is to determine (i) whether there was a processing cost associated with accepting alternating forms relative to correct pronunciations and (ii) whether there was a processing cost to rejecting pre-Sandhi forms in comparison to true mispronunciations. The same method of analysis was used as described above. The first analysis revealed a reduced proportion of shifts to the target in post-Sandhi trials as compared to the correct pronunciation trials late in the processing window for five contiguous epochs (1400–1600; 1600–1800; 1800–2000; 2000–2200; 2200–2400) (*p* < 0.0001) (all contrasts remained significant following Bonferroni correction). Lastly, there was a higher proportion of shifts to the target in pre-Sandhi trials as compared to the mispronunciation trials for three epochs late in the processing window (1600–1800; 1800–2000; 2000–2200) (*p* < 0.0001) (all contrasts remained significant following Bonferroni correction).

An analysis of the time course of word recognition of Sandhi forms reveals a more nuanced picture of how Sandhi forms are processed. In particular, these analyses revealed that post-Sandhi and pre-Sandhi forms were differentially treated as acceptable referents from the onset of the auditory label (following a 200 ms allowance for re-fixation). This difference was observed for the entire duration of the processing window. In addition, time course analyses point to a processing cost linked to Sandhi forms not revealed by naming effects. For post-Sandhi forms, following the offset of the target word, lexical selection appeared to be somewhat weaker than for correct pronunciations late in the processing window. Likewise, target preferences were slightly weaker after the offset of the word for true mispronunciations versus pre-Sandhi forms. These findings demonstrate that the time course of spoken language comprehension differs for Sandhi forms relative to unaltered forms, reflecting a slight processing cost in the time course of lexical selection for Sandhi forms versus non-Sandhi forms. This cost was slightly more evident (i.e., observed a more sustained period) in participants’ acceptance of post-Sandhi forms as labels for targets than in their rejection of pre-Sandhi forms as unacceptable labels for targets.

Finally, our sample comprised a relatively broad age range and as such, we investigated whether responses to Sandhi forms were associated with participants’ age. There were no significant correlations between age in months and naming effects for pre- or post-Sandhi forms or for the other trial types (*p* < 0.5). We conclude that sensitivity to Sandhi in spoken word recognition is relatively stable between 3 and 5 years of age.

## Discussion

The aim of the present study was to investigate whether preschool children could recognize words amidst suprasegmental phonological variation and specifically, whether children could recognize alternating forms attributable to tone Sandhi rules. Preschool children’s abilities to recognize words that had different types of phonological alternations were assessed via a preferential looking paradigm. Familiar target objects were presented to children and accompanied by verbal labels. Labels consisted of four types of words: words altered by Sandhi rules (post-Sandhi forms), words that were unaltered in a Sandhi licensed context (pre-Sandhi forms), unaltered non-Sandhi forms (correct pronunciations) and words that were altered via a tone substitution that was not due to Sandhi (mispronunciations due to a tone substitution). Four primary findings emerged from the study. First, children fixated the target preferentially when encountering post-Sandhi forms as well as correct pronunciations to a similar degree when naming effects were employed as the dependent variable. Second, children fixated the distractor object preferentially when encountering pre-Sandhi forms as well as when encountering true mispronunciations when naming effects were employed as the dependent variable. Third, investigating the temporal dynamics of lexical selection revealed that children’s acceptance and rejection of post-Sandhi and pre-Sandhi forms respectively occurred early, starting shortly after the onset of the first syllable. Fourth, our analyses of the temporal dynamics of lexical selection revealed a slight processing cost associated with post-Sandhi forms relative to correct pronunciations and to a lesser degree, with pre-Sandhi forms relative to true mispronunciations.

The current set of findings provides new insight into our understanding of how young children represent Sandhi forms in memory. Production tasks have revealed that knowledge of tone Sandhi is far from mature in preschool children and children do not attain adult-like maturity until 6 years of age (e.g., Wang, 2011). Our findings suggest that in spoken word recognition, post-Sandhi forms are rapidly mapped onto their referents with comparable facility to correct pronunciations. Likewise, our finding that participants correctly interpreted pre-Sandhi forms as mispronunciations is contrasted with error analyses of Sandhi productions in preschool children demonstrating that 3–5 year old children sometimes substitute pre-Sandhi forms for post-Sandhi forms (Wang, 2011). This suggests that pre- and post-Sandhi forms, although sometimes substituted for one another during speech production, may be representationally ‘intact.’ In other words, children may maintain tacit knowledge that pre-Sandhi forms are not lexically equivalent to post-Sandhi forms. However, it is worth noting that elicitation studies have very different task demands than the present study, specifically in the use of familiar versus novel words, rendering direct comparisons difficult. Further research could investigate whether children make similar production errors when tasked with applying Sandhi rules to familiar words, as compared to novel words.

Although our analyses that focused on aggregate effects (naming effects) revealed that children recognized post-Sandhi forms as correct pronunciations, a more detailed view into the time course of lexical disambiguation demonstrated a potential processing cost to post-Sandhi alternations relative to correct pronunciations. This processing cost appeared late in the time course of lexical disambiguation, after the offset of the second syllable. This pattern of results was not predicted at the outset, but can be theoretically explained by the Multi-Level Cluster Representation model (Zhou and Marslen-Wilson, 1994). Though the presence of a late processing cost for post-Sandhi forms relative to correct pronunciations is incompatible with the underspecification account and canonical account, this pattern of results is consistent with the surface account (see Zhou and Marslen-Wilson, 1997 for a discussion of processing costs of Sandhi forms). According to the Multi-Level Cluster Representation model (Zhou and Marslen-Wilson, 1994), when disyllables are processed in Mandarin, the morphemic representations of constituent syllables are initially activated in the lexicon before the whole compound is activated as speech unfolds. In this way, the processing efficiency of disyllabic compound words arises from the interaction of activation at both levels, i.e., combining information from constituent morphemes and information from whole compound. If this information is convergent, processing is facilitated, but if it is incongruent – as in Sandhi forms – there is a processing cost. As Sandhi forms are not stored in their canonical form according to the surface representation view, the initial speech input does not strongly activate the underlying constituent morpheme. For example, according to the surface view, the initial processing of ‘flour mill’ after Sandhi alternation [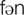
(35) 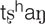
(214)] would not activate ‘flour’ /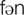
(214)/ and would only activate the second unaltered syllable ‘factory’ /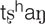
(214)/. This would mean that post-Sandhi equivalent ‘flour mill’ [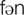
(35) 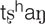
(214)] would be processed less efficiently than the correct pronunciation ‘hill cemetery’[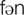
(35) 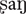
(55)], the latter benefiting from the cumulative activation of both its constituent morphemes ‘grave’/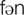
(35)/ and ‘hill’/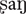
(55)/. In this way, the surface account presupposes a temporal processing cost for Sandhi forms relative to correct pronunciations, evident in our time course analyses. In addition, time course analyses revealed late emergent processing differences for pre-Sandhi forms relative to mispronunciations that were obscured by coarse-grained naming effects analyses. Though processing of pre-Sandhi forms has not been extensively discussed in the prior literature, Zhou and Marslen-Wilson’s (1997) argument that processing differences for post-Sandhi and correct pronunciations stem from multi-level activation in the lexicon (see also Zhou and Marslen-Wilson, 1994), can be extended to account for late emergent processing differences for pre-Sandhi and mispronunciations. In line with the surface view’s postulate that post-Sandhi forms are stored in the lexicon, pre-Sandhi forms would not activate word level representation, i.e., pre-Sandhi equivalent ‘flour mill’ [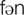
(214) 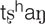
(214)] does not map onto /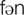
(35) 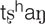
(214)/. However, at the level of the constituent morphemes, both syllables in unaltered canonical form activate individual representations: ‘flour’ /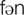
(214)/ and ‘factory’ /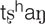
(214)/ respectively. In contrast, the mispronunciation of ‘hill cemetery’ [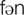
(214) 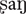
(55)] would only result in the activation of one unaltered constituent ‘hill’ /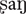
(55)/. This difference in degree of activation could then mean that pre-Sandhi forms are rejected with greater uncertainty than true mispronunciations, thereby resulting in processing differences, a pattern which is evident in our data. To summarize, both aggregate and incremental (time course) analyses preferentially support the surface view of representation.

It is worth noting that in the context of prior literature on processing alternations, it appears that processing cost of post-Sandhi forms relative to non-Sandhi words may not be specific to children nor to tone Sandhi. For example, although adult listeners can compensate for segmental phonological alternations, there is a processing cost to alternations and alternating forms are often processed more slowly than non-alternating (canonical) forms (Gaskell and Marslen-Wilson, 1998; Gow, 2001; Weber, 2001; Kuijpers et al., 2002). Processing costs are particularly high when listeners are deprived of leading contextual cues to lexical identification (Gaskell and Marslen-Wilson, 1998) and when alternations are obligatory versus optional (Otake et al., 1996; Quené et al., 1998). Both of these precipitating conditions were present in the present design and may have potentially increased the cost of processing post-Sandhi forms. Further studies could potentially identify the extent to which leading contextual cues could mitigate the costs of processing Sandhi forms in young children. Similarly, some tone Sandhi rules in Mandarin are optional whilst others are obligatory (Sun, 2006). A comparison of the temporal profile of spoken word recognition for optional versus obligatory tone Sandhi rules (e.g., second tone Sandhi) may shed light on the extent to which processing costs are dependent on the statistical frequency of the Sandhi rule within a language.

In combination, these findings suggest that the apparent under-application of Sandhi rules in children’s productions may not reflect an underdeveloped phonological representation of Sandhi forms, but rather may be due to the effortful nature of integrating Sandhi rules into tone productions. The notion that children may be sensitive to phonological variation in perception, yet sensitivity to the very same source of variation is not reflected in production has been widely documented in early language development. Across multiple tiers of the language code, identification of incorrect forms in a receptive task often precedes the capacity for restoration or repair in productive tasks (see Clark, 1978; Konefal and Fokes, 1984; Smith-Lock and Rubin, 1993; Levy, 1999). In a formalized model of the natural progression from error identification to repair, Karmiloff-Smith (1986) implicates the weighty cognitive burden of identification of erroneous forms in addition to knowing how to repair a word, both of which are needed for accurate word production. In contrast, rejection of erroneous forms in a receptive task alone is much less demanding. This process can hypothetically be enabled by implicit linguistic knowledge that is based on sampling linguistic input and detecting incompatibilities between linguistic input and past experience. By contrast, restoration or repair is only possible within an explicit metacognitive framework that allows for errors to be correctly judged and then appropriately resolved. Differences in children’s negotiation of tone Sandhi across perception and production may well reflect the underlying cognitive demands of error identification versus error identification and repair. It should also be noted that the capacity for repair likely involves an internalization of the underlying rule; we have no evidence in the present study that children have internalized the Sandhi rule. Preferential fixation to the target in response to post-Sandhi forms and to the distractor in response to pre-Sandhi forms could be governed by familiarity alone as post-Sandhi forms are encountered and pre-Sandhi forms are presumably unencountered. Further studies investigating children’s capacities for repair of pre-Sandhi forms could help to determine when children converge upon a rule-based understanding of tone Sandhi.

This brings us to a core question invited by our findings, specifically, the issue of what children store in memory about Sandhi words and the extent to which children harbor tacit knowledge of the phonological rule. It is possible that children sampled in our study were aware of the phonological rule that prescribes Sandhi alternations and that they are well versed in its scope of application. Alternatively, it is possible that children viewed post-Sandhi alternations as correct pronunciations and pre-Sandhi as incorrect pronunciations on the grounds that the former matched normative linguistic input and the latter did not. This possibility would not require rule-based expectations on the part of the learner. The current study does not allow for conclusions to be drawn about children’s application, or even awareness, of the Sandhi rule. Though this question was not directly probed in the current study, it is informative to examine children’s reactions to pre-Sandhi forms included in our study. When children heard pre-Sandhi forms, this should essentially not have been interpreted as a possible word for anything. Instead, these forms were interpreted as a possible label for the distractor (based on children’s early and persistent distractor preferences upon hearing pre-Sandhi forms). If children were indeed aware that two dipping tones could not occur in succession as an obligatory rule, they would be presumably abstain from target or distractor preferences upon hearing these unlicensed forms. The fact that children systematically bound these forms to novel objects leads us to the tentative suggestion that they may not yet have internalized a rule about possible versus impossible Sandhi compounds in the native language. Moreover, time course analyses hint at the possibility that children did not recognize pre-Sandhi forms as a root form of the post-Sandhi forms given that there was no preferential movement toward the target at any phase of the processing window in response to pre-Sandhi forms. Instead, they were treated very similarly to true mispronunciations and linked to distractor objects early in the processing window. Children therefore recognized pre-Sandhi forms as lexically distinct from post-Sandhi forms, but not as lexically impossible. Presumably, later in development, pre-Sandhi forms are discounted as possible words for referents and perhaps linked to the post-Sandhi form via knowledge of the underlying rule. The chronology and precipitants of this transition are not evinced by the current study. Further research with older children could explore responses to pre- and post-Sandhi forms to explore subsequent refinement in children’s awareness of Sandhi rules. This could be accomplished by presenting older children with a visual display containing visual referents that are lexically ambiguous after Sandhi processes. For instance, presenting visual targets for ‘graveyard’ [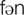
(35) 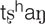
(214)], ‘flour mill’ [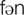
(35) 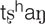
(214)] following a Sandhi alternation and with the pre- and post-Sandhi forms would help to determine whether older children would resist mapping the phonologically illegal form [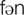
(214) 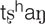
(214)]. In addition, given that third tone Sandhi rule applies across lexical boundaries at a phrase level (see Chen, 2000), further research could also investigate refinement of rule awareness at a phrasal level. Such a comparison could involve comparing participants’ fixation to visual targets corresponding to the non-Sandhi base form ‘bury a horse’ [mai(35) ma(214)] and to the tonally equivalent Sandhi alternation ‘buy a horse’ [mai(35) ma(214)] upon hearing the phrase [mai(35) ma(214)].

As mentioned in the Introduction, theories of spoken language processing must account for the capacity to recover meaning amidst the full range of phonological variation inherent in human speech. This includes suprasegmental variation. There has been considerable interest in adapting prevailing theories of speech perception to accommodate the effects of phonological alternations on adult spoken word recognition (e.g., Gaskell and Marslen-Wilson, 2001; Gow, 2001, 2002; Gow and Im, 2004; Gaskell and Snoeren, 2008; Lahiri and Reetz, 2010). The evidence basis for such models has drawn predominantly from segmental alternations and has examined to a very limited extent the developmental origins of ambiguity resolution arising from alternations. To truly advance generalizable claims in how alternations are processed, evidence from suprasegmental alternations as well as more developmental records of how alternating forms are processed would be valuable additions. According to applications of existing models to tone Sandhi (see Zhou and Marslen-Wilson, 1997), a surface representation account would predict that words are stored in their post-Sandhi form, such that primarily these forms would be activated in lexical retrieval. In contrast, the canonical representation account – advanced by Zhou and Marslen-Wilson (1997) to accommodate tone Sandhi – proposes words are re-written via phonological inference from post-Sandhi forms and stored in their pre-Sandhi form. Finally, the underspecification account would predict that the first syllable of a Sandhi form would be weakly represented with regards to tone, alternating flexibly between a high rising and dipping tone such that both pre-Sandhi and post-Sandhi forms could potentially be activated during word retrieval. The current set of findings demonstrates that post-Sandhi forms were robustly mapped to their targets and pre-Sandhi forms were contrastively mapped to unfamiliar targets. In addition, the time course of lexical disambiguation indicated that post-Sandhi forms are processed similarly to correct pronunciations and that pre-Sandhi forms are processed similarly to mispronunciations. Our findings are therefore most closely consistent with a surface account whereby previously encountered post-Sandhi forms are expected to be successfully linked to targets and pre-Sandhi forms, which are presumably rare in the input, are expected to be rejected as possible labels for familiar words. Our study therefore suggests that the primary unit of representation is the post-Sandhi form. Our findings do not support an underspecification account on the grounds that there was no evidence from time course analyses of co-activation of pre- and post-Sandhi forms at any point in the processing window. Likewise, our findings are not consistent with the canonical view as there was a very similar pattern of target activation – both in terms of strength and immediacy of activation – in response to post-Sandhi and correct pronunciations. As the canonical view posits re-writing of the post-Sandhi form during the second syllable, this account predicts delayed activation for post-Sandhi forms relative to correct forms, which are not subject to regressive interpretation. Moreover, there was immediate rejection of pre-Sandhi forms, not accounted for by the canonical account, which posits the pre-Sandhi form to be the primary unit of representation. Furthermore, the late processing cost associated with post-Sandhi forms (relative to correct pronunciations) and with pre-Sandhi forms (relative to mispronunciations) that was observed in the present study can only be accounted for by the surface view. According to the canonical view, there should be an early processing cost relative to correct pronunciations for post-Sandhi forms before the alternating first syllable is re-written and no processing cost for pre-Sandhi forms. Lastly, in line with the underspecification view, both pre- and post-Sandhi forms should be processed no differently from correct pronunciation words and thus should not be associated with less efficient processing. However, given the relatively small body of research on Sandhi processing in childhood, further corroborative research is needed to more conclusively theorize about the developing lexical representation of Sandhi forms.

An important open question to follow from the current study is the issue of how children learn about tone Sandhi. There have been no developmental investigations of naturalistic linguistic input to infants with regards to tone Sandhi. It remains unclear if adults produce base forms (i.e., two syllables in combination with the dipping tone to preserve tone identity at the syllable level in infant-directed speech) and then override this rule after children’s successful acquisition of the base form. Alternatively, it is possible that disyllables subject to tone Sandhi are introduced as such, and that syllables are later recoded as base forms by language learners. While the order of events remains unclear, it has been suggested that children are likely to learn base forms first followed by Sandhi rules on the grounds that early mastery of post-Sandhi forms could actually obscure mastery of lexical tones (Demuth, 1989). However, the means by which Sandhi is imparted to the language learner and the extent to which Sandhi is present in infant-directed speech awaits further investigation and would potentially be revealed by input analyses of tone language learners.

In summary, this study presents the first investigation of children’s ability to contend with suprasegmental alternations in spoken word recognition. Findings bear on the specificity of early tone representations in relation to contextual cues and reveal a robust ability to negotiate tone Sandhi in pre-school children with minor (and non-catastrophic) temporal processing costs associated with Sandhi forms. In comparison to production studies in early childhood, integration of Sandhi rules in spoken word recognition appears relatively strong. The impact of these findings potentially informs prevailing theories of lexical representation in investigating the early negotiation of suprasegmental phonological alternations in tone languages.

## Author Contributions

TW was involved in conceptualization, data collection, analysis and manuscript preparation. LS was involved in conceptualization, analysis and manuscript preparation.

## Conflict of Interest Statement

The authors declare that the research was conducted in the absence of any commercial or financial relationships that could be construed as a potential conflict of interest.

## Footnotes

1 The other Mandarin tone Sandhi phenomena have received less empirical and theoretical attention (Yang, 2016) and as such, existing studies on children’s and adults’ perception of Sandhi have focused predominantly on the third tone Sandhi rule. In order to interpret our findings with reference to relevant prior studies and theoretical models of Sandhi, we have focused on the third tone Sandhi rule (see Chen, 2000; Lin, 2007 for a discussion of the other Sandhi processes).

2 Due to tonal co-articulation that occurs naturally when producing disyllabic words, the high rising tone does not reach its maximum offset when immediately followed by a high level tone (see Chen, 2000). Therefore, the mean pitch of these two syllables appears different due to effects of tonal co-articulation. This is a natural ‘by-product’ of connected speech. However, the difference in mean pitch is not statistically significant and did not impede recognition of tone identity in our adult sample of native speakers.
